# Educational films for improving perinatal outcomes associated with gestational diabetes in Uganda and India: a cluster randomised trial

**DOI:** 10.1136/bmjgh-2025-022676

**Published:** 2026-04-27

**Authors:** Laura L Oakley, Deepa Ravi, Judith Lieber, Arthur Namara, Yamuna Ana, Helen Coombe, Biswamitra Sahu, Poppy A C Mallinson, Maithili Karthik, Eunice Lobo, Debarati Mukherjee, Nicolas Birk, Lily Hopkins, Iliatha Papachristou Nadal, Janet Seeley, Eugene Oteng-Ntim, Moffat Nyirenda, Sanjay Kinra, Giridhara Rathnaiah Babu

**Affiliations:** 1 Department of Non-Communicable Disease Epidemiology, London School of Hygiene & Tropical Medicine, London, UK; 2 Centre for Fertility and Health, Norwegian Institute of Public Health, Oslo, Norway; 3 Indian Institute of Public Health, Public Health Foundation of India, Bengaluru, India; 4 PHFI Centre for Developmental and Lifecourse Research, Bengaluru, India; 5 MRC/UVRI and LSHTM Uganda Research Unit, Entebbe, Uganda; 6 Medical Aid Films, London, UK; 7 PHFI Centre for Developmental and Lifecourse Research, Bangalore, India; 8 Department of Biostatistics, Harvard University, Harvard T H Chan School of Public Health, Boston, Massachusetts, USA; 9 Division for Care in Long Term Conditions, King’s College London, London, UK; 10 Department of Global Health & Development, London School of Hygiene and Tropical Medicine, London, UK; 11 Department of Women’s Health, Guy's and St Thomas’ NHS Foundation Trust, London, UK; 12 Department of Population Medicine, Qatar University College of Medicine, Doha, Qatar

**Keywords:** Global Health, Obstetrics, Health education and promotion, Maternal health, Diabetes

## Abstract

**Introduction:**

Gestational diabetes mellitus (GDM) is associated with a high risk of adverse perinatal outcomes. We evaluated whether a film-based educational intervention for pregnant women and healthcare providers to improve timely detection and management of GDM could reduce the incidence of adverse perinatal outcomes.

**Methods:**

Two parallel-group cluster randomised trials were conducted in Uganda and India. A package of 7 interconnected culturally-tailored educational films was developed following formative research. 30 government-funded health facilities (clusters) in each country were randomised (1:1) to intervention or control arm. In intervention facilities (15 in each country), films were shared with pregnant women and health professionals; control facilities (15 in each country) received usual care. The outcome was an individual self-reported composite of unplanned caesarean section, stillbirth or neonatal death, or neonatal hospitalisation. Mixed effects models were used in an intention-to-treat analysis with multiple imputation by chained equations to address missing data. Analyses were performed separately for each country; random-effects meta-analysis was used to calculate pooled prevalence ratios (PRs). Data analysts were blinded to group allocation, but participants, facility and trial staff were not.

**Results:**

In Uganda, 5495 women were screened between May 2021 and April 2022, and 5102 (92.8%) participated in the trial. In India, 12 045 women were screened between July 2021 and January 2022, and 10 899 (90.5%) participated. Loss to follow-up was 31.7% in Uganda and 12.1% in India. In Uganda, the prevalence of the composite adverse perinatal outcome was 19.7% in the control arm and 19.8% in the intervention arm (PR 1.00, 95% CI 0.87 to 1.14). For India, the prevalence was 29.5% and 30.6%, respectively (PR 1.04, 95% CI 0.96 to 1.11). The pooled PR across both countries was 1.03 (95% CI 0.97 to 1.10).

**Conclusions:**

A film-based intervention did not reduce the incidence of adverse perinatal outcomes associated with GDM. Future evaluations should assess educational films delivered alongside more intensive intervention components.

**Trial registration numbers:**

NCT03937050, ISRCTN96432637.

**WHAT IS ALREADY KNOWN ON THIS TOPIC**

**WHAT THIS STUDY ADDS**

- To our knowledge, this is the first evaluation of a film-based intervention for pregnant women and health professionals aimed at improving the detection, management and associated complications of GDM.
- Across two large cluster randomised trials conducted in Uganda and India, we found no evidence that educational films reduced the incidence of a self-reported composite of unplanned caesarean section, stillbirth or neonatal death, or neonatal hospitalisation.

**HOW THIS STUDY MIGHT AFFECT RESEARCH, PRACTICE OR POLICY**

- A brief film-based intervention alone may be insufficient to impact on GDM-associated adverse perinatal outcomes, but could complement more intensive, multicomponent approaches given its low cost and scalability.

## Introduction

 Gestational diabetes mellitus (GDM) is associated with an increased risk of adverse perinatal outcomes, including stillbirth and birth complications.^1^,^5^ The global prevalence of GDM is estimated to be 14%, with the prevalence rising over time.^6^ A recent meta-analysis suggests that 13% of pregnancies in India are complicated by GDM.^7^ Fewer estimates are available for Uganda, but a large study conducted in antenatal clinics reported that 8.5% of women had hyperglycaemia in pregnancy.^8^

As the prevention of GDM is challenging, the focus has largely been on improving the detection and management of the condition. Appropriate management of GDM can reduce perinatal complications and the risk of longer-term complications for both the woman and offspring.^6 9 10^

Over recent years, there has been increasing interest in the use of film to promote health education messages.^11 12^ Film-based interventions are low-cost and sustainable and are ideal for addressing the growing burden of chronic disease, particularly in low-income and middle-income country (LMIC) settings where they can overcome barriers associated with low literacy. A recent review of trials of digital interventions, some of which included brief film components, found evidence that they can increase participation in cancer screening.^13^ A number of trials have evaluated interventions for women at risk of GDM, focusing on promoting general health education, evaluating different screening approaches, and lifestyle and treatment interventions for those diagnosed with GDM. To our knowledge, no film-based educational interventions targeted at this population have been evaluated.

The GUIDES (Gestational diabetes in Uganda and India: Design and Evaluation of Educational Films for Improving Screening and Self-management) trial was designed to develop and evaluate a package of short educational films designed to improve awareness, screening and management of GDM in two LMIC settings: Uganda and India. The intervention was designed to be delivered to pregnant women and health professionals at the health facility level. The primary outcome for this trial was the proportion of pregnant women screened for GDM,^14^ which is reported elsewhere. We hypothesised that the intervention could reduce GDM-related adverse perinatal outcomes through two complementary mechanisms. First, improved detection and management of GDM could lead to better detection and more timely treatment. Second, we hypothesised that promotion of healthier lifestyle behaviours among pregnant women more broadly could lead to improved maternal glucose levels. Given the strong and continuous association between maternal glucose levels and adverse perinatal outcomes,^2^ including below diagnostic thresholds, these pathways were expected to reduce perinatal risk and, for some women, prevent progression to GDM.

This manuscript reports the results of the main secondary outcome of the GUIDES trial: a composite of unplanned caesarean section (CS), stillbirth or neonatal death, or neonatal hospitalisation.

## Methods

### Design and setting

We conducted two parallel-group cluster randomised controlled trials (RCTs) in Uganda and India. The protocol has been published previously,^14^ but the study design is summarised here. In each country, study teams recruited 30 government-funded health centres providing maternity care and recording a minimum of 200 births per year. The Uganda trial was conducted in Central Uganda, covering a mixture of urban and rural areas. The India trial was conducted in urban Bengaluru in the southern state of Karnataka. Randomisation was at the cluster level with a 1:1 ratio and took place after health centre recruitment. Covariate constrained randomisation was performed by an independent statistician separately for each trial. The Stata command *cvcrand* was used to generate 100 000 random allocation sequences. For each sequence, an imbalance score was calculated based on facility size, facility level and urban/periurban or rural setting (Uganda only). A subset of 2500 permissible allocations was generated by retaining allocations with imbalance scores in the lowest 2.5th percentile. All retained allocations demonstrated good balance across the selected covariates. Finally, one allocation was randomly selected as the definitive randomisation scheme. Due to the trial design, group allocation could not be concealed from participants, health professionals and trial staff. Data analysts were blinded to allocation status.

We followed the Consolidated Standards of Reporting Trials extension for cluster randomised trials.^15^

### Participant recruitment and consent

Pregnant women registered at participating health facilities were eligible for the trial if they were ≥18 years old, <32 completed gestational weeks at recruitment, and available for follow-up. In Uganda, fieldworkers visited antenatal care (ANC) waiting areas in participating facilities, distributed patient information sheets (PIS), recruited and obtained written informed consent in person. In India, COVID-19-related pandemic restrictions meant that eligible pregnant women in India were identified using the local mother and child health database. Fieldworkers contacted potentially eligible women by telephone and sent the PIS electronically; informed consent was obtained via e-consent. Recruitment materials were available in Luganda and English for the Uganda trial, and in Dhakani, Kannada and English for the India trial. Participants were recruited in Uganda between May 2021 and April 2022, and in India between July 2021 and January 2022.

### Data collection

Participants completed a baseline questionnaire at enrolment. In Uganda, this was completed in person with fieldworkers; in India, the baseline questionnaire was completed by telephone. In both countries, follow-up questionnaires were administered by telephone at approximately 32 weeks of pregnancy (FU1) and 6 weeks after the expected date of delivery (FU2). Perinatal outcome was assessed in the postnatal questionnaire, although a short set of questions was asked at FU1 where the pregnancy had already ended. Due to the COVID-19 disruption to the trial in Uganda, some women who missed FU1 were administered a modified FU2 questionnaire assessing perinatal outcome. Data collection was completed in April 2023 in Uganda and in May 2023 in India. Data were entered contemporaneously by fieldworkers to a custom-built secure app in India and RedCap in Uganda. All trial staff were trained in study procedures and good clinical practice.

### Intervention and comparison

We partnered with Medical Aid Films (MAF) (*https://www.medicalaidfilms.org/*) to produce an interconnected package of educational films about gestational diabetes for pregnant women and health professionals. The films were developed following extensive formative research with pregnant women, women with a previous GDM diagnosis and stakeholders,^16 17^ and the full intervention development process is described elsewhere.^14^ Each package consisted of the following: (a) a film for pregnant women to raise awareness of GDM; (b) a set of four films for pregnant women diagnosed with GDM, focusing on improving confidence and skills for self-management and (c) a film for doctors and nurses, improving skills and knowledge regarding optimal GDM screening and management. Each film was between four and 10 min in length. The films for pregnant women with GDM encouraged women to take a proactive role in managing their condition through goal setting for behaviour change, with tips on healthy eating and appropriate physical activity. The films also covered how to store and inject insulin, a visual demonstration of blood sugar testing, and how to recognise and treat hypoglycaemia. Film content was adapted to reflect the specific cultural and clinical context (eg, dietary advice refers to locally available food; clinical advice reflects local/national guidelines). Films were subtitled, and audio versions were available in Dhakani and Kannada in India, Luganda in Uganda, and also a locally accented English voiceover.

In Uganda, television screens in ANC waiting rooms played the introductory film continuously, and videos for women with GDM were shown at dedicated facility-based sessions. Due to COVID-19 pandemic restrictions in India, films for pregnant women were shared with participants via WhatsApp as the primary mode of viewing. All participants in the India trial reported at baseline that they had access to a smartphone, and the use of WhatsApp is widespread. In both countries, the films for doctors and nurses were played at regular facility staff meetings.

Health facilities allocated to the control arm followed usual care practices. Usual care differed between the two country settings. In India, universal screening for GDM is recommended,^18^ but evidence suggests that practice is variable.^19^ In a 2014 survey of doctors in Bengaluru, only 50% demonstrated good knowledge of GDM management.^20^ In Uganda, although national guidelines recommend screening in the presence of risk factors,^21^ formal data are limited and evidence suggests that screening is largely non-existent and management highly variable.^22^

### Outcomes

The outcome for the analysis reported here (described as secondary outcome 1 in our study protocol)^14^ was a composite outcome indicating any of the following: birth by unplanned CS, stillbirth or neonatal death, or neonatal hospitalisation. Neonatal hospitalisation was defined as either admission to a neonatal intensive care unit (NICU) or infant hospitalisation during the first 28 days. The use of a composite perinatal outcome is consistent with previously conducted trials on GDM.^9 10 23^

### Sample size

Initial sample size calculations for the GUIDES trial were performed according to the primary trial outcome of GDM screening and diagnosis. For the composite outcome reported here, which was a secondary outcome of the GUIDES trial, no published estimates were available for the likely prevalence. We therefore assumed a baseline prevalence of 35% for the composite outcome. This was informed by a previous trial including women with mild GDM only,^10^ and also data on CS delivery (24%), and neonatal and perinatal mortality (both <2.5%) from the National Family Health Study 4 (2015–2016) reported for Karnataka state, India,^24^ together with an estimate that approximately 10% of neonates need additional care at birth.^25^ In the absence of relevant data, an indicative intracluster correlation (ICC) of 0.01 was used. This was loosely informed by a cluster randomised trial of pregnancy outcomes in low-income country settings that applied an ICC of 0.005 for a composite maternal and neonatal outcome. Given the higher prevalence of our composite outcome, we assumed a slightly higher degree of clustering. We did not consider variability in cluster size at this stage.

Using these assumptions, we calculated that a sample size of 5935 women across 30 clusters (average cluster size 200 births) would be required for each trial to detect a reduction from 35% to 30%, assuming 80% power at a 5% significance level, using an ICC of 0.01.

### Statistical analysis

All analyses adhered to our Statistical Analysis Plan (SAP).^26^ Data were analysed at the individual level with separate analyses for each country. Our primary analysis was intention-to-treat. We used a logistic mixed-effects model with a random intercept for health centre to account for clustering, as pre-specified in our SAP, and fixed effects for trial arm and the prespecified cluster-level variables, which included facility level, facility size, and, for Uganda only, urban versus rural setting. A random intercept structure was decided a priori, instead of a more complex specification such as random slope, because the same intervention was implemented across all intervention sites, and we assumed that its effect would not vary substantially across clinics. We calculated model-based prevalence risks by trial arm, and the prevalence difference and prevalence ratio (PR). As specified in our SAP, we also estimated models adjusted for key a priori individual-level confounders: age, parity and education level. All analyses excluded participants whose pregnancies lasted ≤6 completed months.

We imputed missing data by imputation by chained equations (MICE) using the Stata *mi impute* command. Each incomplete variable was imputed using a univariate model appropriate to its distribution. The imputation model included all covariates in our primary analysis and additional auxiliary variables associated with missingness. We derived 20 imputed datasets, with estimates combined using Rubin’s rules. Imputed results are shown as the primary analysis.

In additional analyses, we separately assessed each of the individual components that contributed to the composite outcome.

We also conducted complete case analyses and per-protocol analyses (using self-reported viewing data). Finally, in a post hoc sensitivity analysis, we adjusted for individual-level covariates that were unbalanced at baseline.

We pooled estimates for the main composite outcome across the trials using a random-effects meta-analysis with inverse variance weighting of individual-country results.

Analyses were performed using Stata V.18 (StataCorp).

### Patient and public involvement

Patients and the public were not directly involved in designing the research question, outcome measures or overall study design. However, formative public and stakeholder engagement informed the development of the intervention. In addition, an accompanying process evaluation gathered feedback from pregnant women and stakeholders on their experiences and perceptions of the study.

## Results

**Figure 1:**
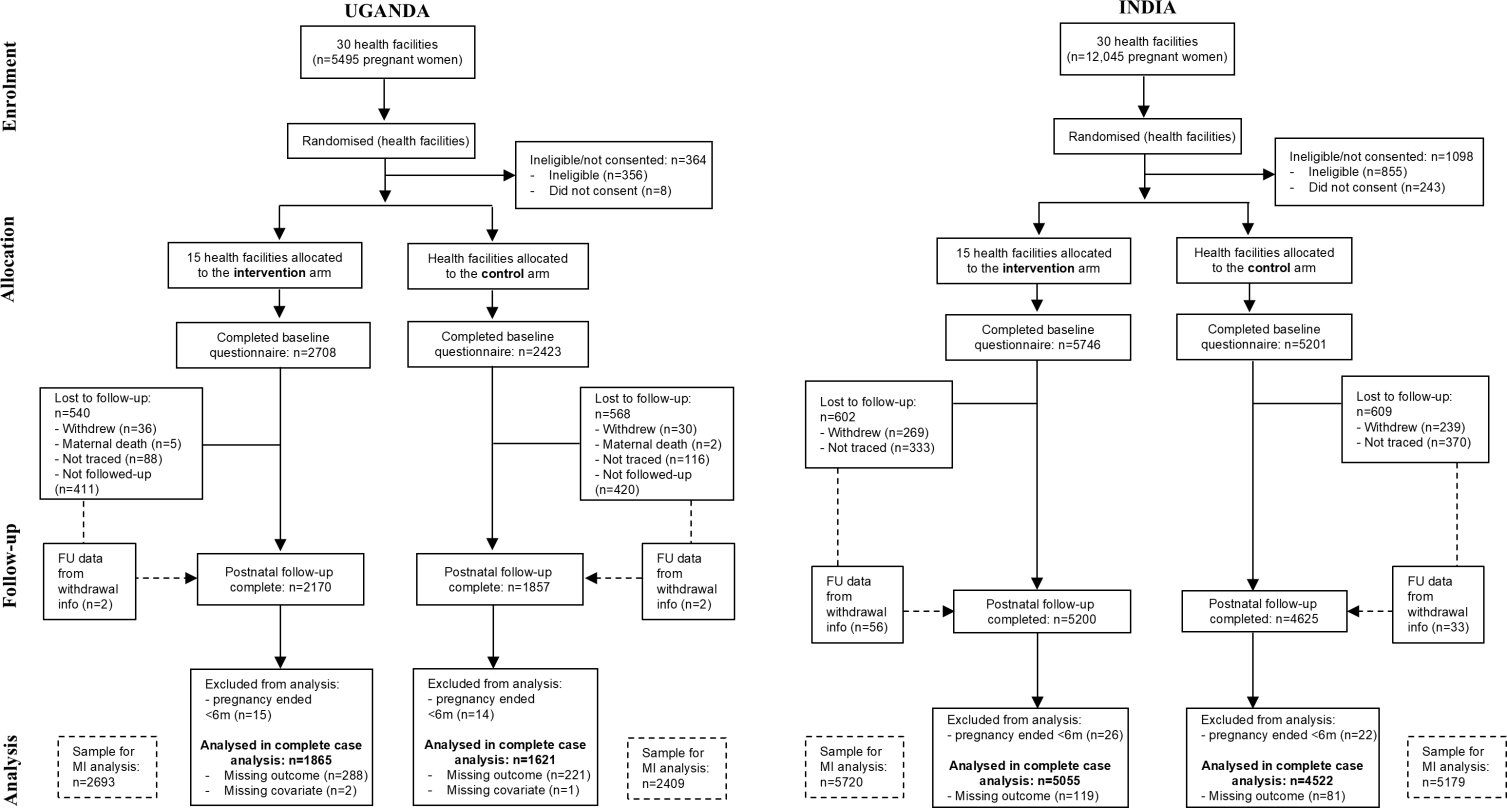
CONSORT flow diagram. CONSORT, Consolidated Standards of Reporting Trials; FU, follow-up.

All participating health centres (clusters) adhered to their original allocation and were included in the analysis. The characteristics of participating health centres are presented in online supplemental table 1. In Uganda and India, 5495 and 12 045 pregnant women were screened for inclusion, respectively (figure 1). Of these numbers, 5131 (93.4%) and 10 947 (90.9%) were eligible for the study, consented and completed the baseline questionnaire. A small number of participants whose pregnancies lasted less than six completed months were excluded from the analysis (n=29 in Uganda, n=48 in India). The final number of participants eligible for inclusion in the analysis was 5102 (92.8%) in Uganda and 10 899 (90.9%) in India. In Uganda, the mean gestational age at baseline was 23.4 weeks (SD 5.9), and in India, it was 24.4 weeks (SD 3.3). At baseline, 97 (0.9%) of women in India reported having already been diagnosed with GDM in their current pregnancy, but no women reported this at baseline in the Uganda trial.

The characteristics of all participants who completed the baseline questionnaire and were included in the analysis are presented by trial arm and country in table 1, with participants by health facility presented in online supplemental tables 2 and 3. Compared with the India trial, participants in the Uganda trial were younger, less likely to be married, had lower levels of education and were more likely to be in paid employment. Participants in the India trial were more likely to be nulliparous, more likely to have perceived income insufficiency and to have had their first ANC visit earlier.

**Table 1:** Characteristics of trial participants

	Uganda (N=5102)		India (N=10 899)	
**Characteristic**	**Control** **n=2409** **No. (%**)	**Intervention** **n=2693** **No. (%**)	**Control** **n=5179** **No. (%**)	**Intervention** **n=5720** **No. (%**)
Age (years)				
<20	322 (13.4)	369 (13.7)	287 (5.5)	297 (5.2)
20–24	955 (39.6)	1149 (42.7)	1912 (36.9)	2113 (36.9)
25–29	661 (27.4)	691 (25.7)	1919 (37.1)	2171 (38.0)
30–34	300 (12.5)	321 (11.9)	833 (16.1)	904 (15.8)
≥35	171 (7.1)	163 (6.1)	228 (4.4)	235 (4.1)
Marital status				
Married	1525 (63.3)	1566 (58.2)	5179 (100.0)	5720 (100.0)
Cohabiting	809 (33.6)	1041 (38.7)		
Not in a partnership	75 (3.1)	86 (3.2)		
Highest level of education (own)				
None or primary school	882 (36.6)	978 (36.3)	389 (7.5)	484 (8.5)
Secondary or high school	1324 (55.0)	1525 (56.6)	3394 (65.5)	3704 (64.8)
Graduate or postgraduate	203 (8.4)	190 (7.1)	1396 (27.0)	1532 (26.8)
Highest level of education (partner)				
None or primary school	344 (14.3)	355 (13.2)	964 (18.6)	1120 (19.6)
Secondary or high school	1160 (48.2)	1244 (46.2)	2893 (55.9)	3277 (57.3)
Graduate or postgraduate	292 (12.1)	332 (12.3)	849 (16.4)	809 (14.1)
Don't know	538 (22.3)	676 (25.1)	473 (9.1)	514 (9.0)
N/a–no partner	75 (3.1)	86 (3.2)		
Perceived sufficiency of household income				
Yes, it allows me/us to build savings	104 (4.3)	159 (5.9)	338 (6.5)	354 (6.2)
Yes, it allows me/us to save a little	606 (25.2)	601 (22.3)	370 (7.1)	396 (6.9)
Yes, it is just enough	925 (38.4)	1120 (41.6)	1263 (24.4)	1330 (23.3)
No, I/we must use savings	467 (19.4)	505 (18.8)	2323 (44.9)	2657 (46.5)
No, I/we must borrow	307 (12.7)	308 (11.4)	885 (17.1)	983 (17.2)
Occupation before pregnancy				
Homemaker/unemployed/student	1378 (57.2)	1604 (59.6)	4165 (80.4)	4608 (80.6)
In paid employment	1031 (42.8)	1089 (40.4)	1014 (19.6)	1112 (19.4)
First ANC visit in months, mean (SD)	3.57 (1.60)	3.53 (1.66)	2.44 (0.50)	2.42 (0.49)
Parity				
0	833 (34.6)	980 (36.4)	3313 (64.0)	3678 (64.3)
1	660 (27.4)	759 (28.2)	1431 (27.6)	1602 (28.0)
2	433 (18.0)	427 (15.9)	403 (7.8)	428 (7.5)
≥3	481 (20.0)	525 (19.5)	32 (0.6)	12 (0.2)
Multiple birth				
No	1804 (98.7)	2096 (98.1)	4544 (99.4)	5091 (99.5)
Yes	23 (1.3)	40 (1.9)	26 (0.6)	27 (0.5)
GDM introduction film viewed ≥1 times				
Yes	n/a	2213 (94.9)	n/a	5272 (97.0)
No	n/a	112 (4.8)	n/a	118 (2.2)
Don't know	n/a	7 (0.3)	n/a	43 (0.8)

ANC, antenatal care; GDM, gestational diabetes mellitus; n/a, not available.

A comparison between participants with and without follow-up data is presented in online supplemental table 4. In the Uganda trial, nearly one-third of participants were lost to follow-up or had missing data (intervention arm 30.7%, control arm 32.6%). Participants lost to follow-up were slightly younger, less likely to be married and had lower education and parity. Loss to follow-up was lower in India: 11.6% in the intervention arm and 12.7% in the control arm. In India, participants lost to follow-up tended to be of lower parity and had initiated ANC earlier.

### Primary analysis

**Figure 2:**
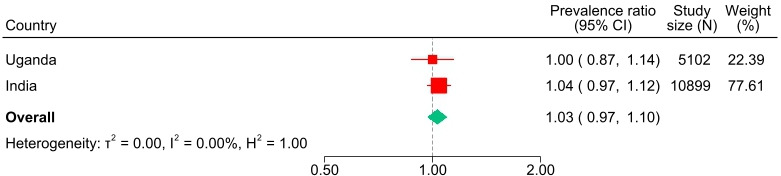
Meta-analysis of composite adverse perinatal outcomes.

In the main preplanned analysis using multiple imputation and controlling for all a priori covariates, the model-based prevalence risk in Uganda was 19.8% in the intervention arm and 19.7% in the control arm (PR 1.00, 95% CI 0.87 to 1.14) (table 2). In India, the prevalence risk was 30.6% in the intervention arm and 29.5% in the control arm (PR 1.04, 95% CI 0.96 to 1.11). The pooled PR across both countries was 1.03 (95% CI 0.97 to 1.10) (figure 2). Repeating the analysis without adjustment for a priori individual-level covariates (age, parity, education) did not change the estimates (online supplemental table 5, online supplemental figure 1).

**Table 2:** Preplanned analyses for perinatal composite outcome and individual perinatal component outcomes

	Uganda					India				
	**Prevalence control (95% CI**)	**Prevalence intervention (95% CI**)	**Prevalence difference (95% CI**)	**Prevalence ratio (95% CI**)	**ICC**	**Prevalence control (95% CI**)	**Prevalence intervention (95% CI**)	**Prevalence difference (95% CI**)	**Prevalence ratio (95% CI**)	**ICC**
Perinatal composite outcome	19.73 (17.97 to 21.67)	19.80 (18.02 to 21.75)	0.07 (−2.62 to 2.76)	1.00 (0.87 to 1.14)	0.000	29.54 (27.91 to 31.26)	30.57 (29.02 to 32.21)	1.03 (−1.25 to 3.32)	1.04 (0.96 to 1.11)	0.002
Unplanned CS	9.03 (7.62 to 10.70)	8.43 (7.20 to 9.87)	−0.60 (−2.43 to 1.22)	0.93 (0.74 to 1.18)	0.001	22.68 (21.36 to 24.08)	22.97 (21.70 to 24.31)	0.29 (−1.56 to 2.14)	1.01 (0.93 to 1.10)	0.001
Stillbirth or neonatal death	4.29 (3.38 to 5.43)	5.69 (4.74 to 6.83)	1.40 (−0.03 to 2.83)	1.33 (0.94 to 1.73)	0.000	4.80 (4.15 to 5.55)	5.72 (5.08 to 6.45)	0.92 (−0.06 to 1.91)	1.19 (0.99 to 1.44)	0.002
NICU or infant hospitalisation	9.19 (7.34 to 11.51)	8.45 (5.96 to 11.98)	−0.74 (−2.99 to 1.51)	0.92 (0.67 to 1.16)	0.000	4.83 (3.72 to 6.26)	5.00 (3.63 to 6.89)	0.17 (−0.83 to 1.17)	1.04 (0.69 to 1.57)	0.002

All models are intention-to-treat, use multiple imputation and are adjusted for a priori individual-level and cluster-level covariates ICC.

CS, caesarean section; ICC, intraclass correlation coefficient; NICU, neonatal intensive care unit.

### Additional analyses

In Uganda, 94.9% (n=2213) of participants in intervention facilities reported that they had seen the GDM introduction film at least once, and the corresponding percentage in India was 97.0% (n=5272). The results of the per-protocol analysis were almost identical to the primary analysis (online supplemental table 5, online supplemental figure 2).

In both Uganda and India, results from the complete case analysis were virtually identical to those obtained in the primary analysis (online supplemental table 5). The complete case pooled PR across both countries was the same as the primary pooled PR (online supplemental figure 3).

Repeating the analyses with additional adjustment for individual-level covariates that were unbalanced at baseline did not markedly impact the estimates (online supplemental table 5, online supplemental figure 4). A summary analysis at the cluster level was consistent with the results from the primary analysis (online supplemental table 6).

### Individual perinatal outcomes

In Uganda, the model-based prevalence of unplanned CS delivery was 8.4% and 9.0% for the intervention and control arm, respectively (PR 0.93, 95% CI 0.74 to 1.18) (table 2). In India, the model-based prevalence of unplanned CS was 23.0% in the intervention arm and 22.7% in the control arm (PR 1.01, 95% CI 0.93 to 1.10).

The prevalence of stillbirth or neonatal death was similar in both Uganda and India, ranging between 4% and 6%, and although it was slightly higher in the intervention group, the observed differences were compatible with chance (Uganda PR 1.33, 95% CI 0.94 to 1.73; India PR 1.19, 95% CI 0.99 to 1.44).

Neonatal hospitalisation was higher in Uganda compared with India (Uganda prevalence intervention 8.5%, prevalence control 9.2%, PR 0.92, 95% CI 0.67 to 1.16; India prevalence intervention 5.0%, prevalence control 4.8%, PR 1.04, 95% CI 0.69 to 1.57).

## Discussion

We hypothesised that a package of interconnected educational films aimed at improving the timely detection and management of GDM in Uganda and India could reduce the incidence of a GDM-related composite perinatal outcome comprised of unplanned CS, stillbirth or neonatal death, or neonatal hospitalisation. Approximately 23% of participants in our Uganda trial experienced at least one of the composite adverse perinatal outcomes, and approximately 30% in India. Our findings do not support a strong positive impact of the intervention on adverse perinatal outcomes in the trial settings.

The GUIDES study included two of the largest trials ever conducted of GDM interventions, recruiting over 15 000 pregnant women in total. The study was conducted in two settings with differences in baseline screening practices, therefore increasing the generalisability of findings.

We evaluated the use of short educational films for pregnant women and health professionals. This intervention medium is of increasing interest, ideally suited for mass delivery and requiring minimal supporting infrastructure. There is a lack of evidence regarding the effectiveness of film-based interventions in public health; therefore, this study helps to address an important knowledge gap.

The intervention films were developed alongside MAF, an organisation that has established expertise in producing films designed to improve the health and well-being of women and children worldwide. The films were based on extensive formative development work and refined iteratively to ensure they were culturally acceptable and addressed barriers and facilitators to GDM screening and healthy lifestyles in pregnancy.

We used a composite perinatal outcome, as has been used in similar trials.^9 10 23^ Composite outcomes are commonly used in large obstetric trials because serious clinical outcomes, such as mortality, are often rare.^27^ We ensured that our composite outcome included components relating to maternal and neonatal health. Due to practical constraints—specifically, reliance on self-reported data without the ability to validate responses against medical records—we restricted the components to those for which there is evidence of good maternal recall,^28 29^ and we used question wording from established questionnaires.^30^ Other GDM-relevant outcomes such as birth trauma and neonatal hypoglycaemia were not considered feasible to collect via self-report. In our analysis, unplanned caesarean birth was substantially more prevalent than the other components, meaning that the overall composite result was largely driven by this outcome. To aid interpretation, we therefore also presented results for each individual component outcome. While the prevalence of perinatal death was slightly higher in the intervention group, there is no clear theoretical reason how the intervention could have increased perinatal death and the observed differences were small and uncertain. Therefore, this finding should be interpreted with caution. Reassuringly, the final trial prevalence of the composite outcome in India was almost identical to the estimate used in our sample size calculation.

The global COVID-19 pandemic caused severe disruption to the GUIDES study. Trial set-up was underway when the pandemic started to spread to India and Africa, and trial activity had to be paused as both countries began instituting intermittent restrictions. The pandemic caused significant disruption to ANC delivery in both countries. Local restrictions, as well as the fear of infection, led to a decrease in women attending ANC.^31^,^33^ Furthermore, in Bengaluru, women were prohibited from waiting inside clinics. We instituted protocol amendments for the India trial to enable e-recruitment and e-consent, with recruitment via a central administrative database of ANC attendees. In India, the trial was adapted to enable the sharing of intervention films via social media, possible due to 100% smartphone coverage in our study population and the widespread use of WhatsApp. In Uganda, sharing of the films via social media was not feasible due to the lack of comprehensive smartphone coverage (17.8% of participants reported that they did not have access to a smartphone). The field team in Uganda was redeployed at the height of the pandemic, resulting in a slower pace of recruitment and intermittent pauses in data collection. We did not achieve our target in Uganda (5102 women recruited vs 5935 intended), meaning the trial there was underpowered and results should be interpreted with caution. In contrast, we exceeded the sample size target in India, providing greater certainty than initially expected. The Uganda trial also experienced higher loss to follow-up (31.7% vs 12.1% in India). We addressed loss to follow-up using multiple imputation under the assumption that data were missing at random (MAR). Most missing outcome data appeared to arise from practical challenges in ensuring timely follow-up during the COVID-19 pandemic rather than selective dropout related to outcome status, and outcome prevalences in Uganda were broadly comparable to those in India, except for caesarean delivery which was expected to differ. The distribution of key risk factors was also similar between participants with and without missing outcome data, supporting the plausibility of the MAR assumption. Our MICE model included all variables from the main analysis as well as additional auxiliary variables associated with both missingness and the outcome. Results from the complete-case analyses were very similar, suggesting that our findings were not sensitive to how missing data were handled. We assumed an ICC of 0.01 for the sample size calculation, but the observed ICCs were very low (range 0.000 to 0.002), indicating minimal clustering. This means that statistical power would likely have been at least as high as planned if our intended sample size had been met.

Previous trials have assessed the impact of lifestyle and other non-pharmacological interventions on perinatal outcomes associated with GDM,^34 35^ but to our knowledge, this study is the first to evaluate the impact of a film-based educational outcome on adverse perinatal outcomes associated with GDM. A series of Cochrane reviews of exercise and/or dietary interventions in pregnancy did not find evidence to support a reduction in GDM, though the evidence on caesarean delivery, perinatal mortality and NICU admission was mixed.^36^,^38^

Although the films we evaluated in the GUIDES trial focused primarily on GDM screening and management, advice on healthy lifestyles during pregnancy was incorporated. For example, the introductory film about GDM screening shared tips about eating well in pregnancy and keeping active during pregnancy. We hypothesised that the intervention could result in a decreased incidence of adverse perinatal outcomes through two different pathways: First, by increasing the detection of GDM and improving the management of women with GDM, and second, by encouraging healthy behaviour among all pregnant women.

Despite previous evidence that film can be an effective format for health education and supporting behaviour change,^11 39^ our results do not support a major impact when used alone. This analysis is based on clinical outcomes, which are heavily influenced by factors such as pre-existing maternal morbidity and healthcare services. A positive impact of the films may have been detected if we had focused on health behaviour as a primary outcome instead. Our per-protocol analysis was based on participants who viewed at least the introductory film. There was no meaningful difference between the estimates from the intention-to-treat analysis and the per-protocol analysis, reflecting the high intervention penetrance: 94.9% of intervention participants in Uganda and 97.0% in India reported watching at least one film, with similarly high proportions (94.3% in Uganda, 99.1% in India) among women who reported a GDM diagnosis. We did not collect information on repeat viewing, and it is possible that most intervention participants only watched a film once, which may have been insufficient to generate an effect. Due to necessary COVID-19 adaptations in India, the intervention films were shared remotely in India as the primary viewing mode rather than played in clinic settings as originally planned and as implemented in Uganda. However, early findings from the process evaluation suggest that some participants in the India trial did view the films in clinic settings, likely at the end of the trial once pandemic restrictions were lifted. Despite the different approaches, a similarly high proportion of intervention participants reported watching at least one film. Both approaches have potential disadvantages: in busy clinics, distractions such as general noise or the need to take care of accompanying children may have prevented women from absorbing the key messages of the films. Viewing films on a smartphone (as in India) in a busy home environment may have affected a participant’s ability to give the films their full attention, though remote viewing also allows for repeat viewing which may increase the impact of health education messages. Overall, sharing the films in clinic settings maximises inclusivity and represents a low burden for pregnant women, health professionals and health clinics. Additionally, women diagnosed with GDM who view the GDM-specific films in a clinic setting (rather than remotely) may have benefitted from in-person support from health professionals and peers. A full process evaluation of the GUIDES trial is currently in progress^40^ and will explore potential mechanisms and will provide further insights into why the intervention did not impact perinatal outcomes.

## Conclusions

This study aimed to evaluate whether an intervention based on short educational films—easily scalable and resource-light—could be sufficient to improve outcomes. Our results do not support a beneficial impact of the GUIDES films alone in reducing the incidence of GDM-related adverse perinatal outcomes in Uganda and India. Further research should explore whether film-based educational interventions could be used to augment the delivery of more intensive intervention components in order to address the growing burden of GDM and associated healthcare costs in LMIC settings.

## Supplementary material

10.1136/bmjgh-2025-022676 online supplemental file 1

10.1136/bmjgh-2025-022676 online supplemental file 2

## Data Availability

Data are available on reasonable request.
