# Supplementary material for: Educational films for improving perinatal outcomes associated with gestational diabetes in Uganda and India: a cluster randomised trial
Source: BMJ Glob Health. 2026 Apr 27;11(4):e022676. doi: 10.1136/bmjgh-2025-022676 (PMC13141209; doi:10.1136/bmjgh-2025-022676)
Supplement: online supplemental file 1 [file bmjgh-11-4-s001.pdf]

SUPPLEMENTARY FILE

Contents

List of intervention films used in the GUIDES project 2
Supplemental Table 1. Characteristics of health facilities by trial arm, Uganda (N=30) 3
Supplemental Table 2. Characteristics of health facilities by trial arm, India (N=30) 3
Supplemental Table 3. Participants by health facility, trial arm and country 4
Supplemental Table 4. Baseline characteristics of participants lost to follow-up 5
Supplemental Table 5. Additional pre-planned analyses for perinatal composite outcome 7
Supplemental Table 6. Cluster level summary analysis 8
Supplemental Figure 1. Meta analysis without adjustment for *a priori* individual-level confounders 9
Supplemental Figure 2. Meta analysis for per protocol analysis 9
Supplemental Figure 3. Meta analysis for complete case analysis 9
Supplemental Figure 4. Meta analysis with additional adjustment for individual-level covariates unbalanced at baseline 10

List of intervention films used in the GUIDES project

UGANDA FILMS		
**Film name**	**Film title**	**Target audience**
Intro	What is gestational diabetes? Uganda English with subtitles	Pregnant women receiving antenatal care at intervention health facilities
Film One	Introduction to gestational diabetes Uganda English with subtitles	Pregnant women diagnosed with GDM receiving antenatal care at intervention health facilities
Film Two	Making healthy lifestyle choices in pregnancy Uganda English with subtitles	
Film Three	Treatment for gestational diabetes Uganda English with subtitles	
Film Four	Coping with a diagnosis of gestational diabetes Uganda English with subtitles	
Doctors	Screening and Management of Gestational Diabetes: A film for doctors Uganda English with subtitles	Doctors caring for pregnant women at intervention health facilities
Nurses	Screening and Management of Gestational Diabetes: A film for Nurses and Midwives Uganda English with subtitles	Nurses and midwives caring for pregnant women at intervention health facilities

INDIA FILMS		
**Film name**	**Film title**	**Target audience**
Intro	What is gestational diabetes? India English with subtitles	Pregnant women receiving antenatal care at intervention health facilities
Film One	Introduction to gestational diabetes India English with subtitles	Pregnant women diagnosed with GDM receiving antenatal care at intervention health facilities
Film Two	Making healthy lifestyle choices in pregnancy India English with subtitles	
Film Three	Treatment for gestational diabetes India English with subtitles	
Film Four	Coping with a diagnosis of gestational diabetes India English with subtitles	
Doctors	Screening and Management of Gestational Diabetes: A film for doctors India English with subtitles	Doctors caring for pregnant women at intervention health facilities
Nurses	Screening and Management of Gestational diabetes: A film for Nurses and Midwives India English with subtitles	Nurses and midwives caring for pregnant women at intervention health facilities

The GUIDES films can be viewed via the Medical Aid Films website https://www.medicalaidfilms.org/

Supplemental Table 1. Characteristics of health facilities by trial arm, Uganda (N=30)

		Control (n=15)	Intervention (n=15)
**Facility level**	**Level 3 (sub-county)**	11	12
	**Level 4 (county)**	4	3
**Approximate births per year**	**<500**	7	7
	**500-999**	3	4
	**1000-1499**	3	2
	**≥1500**	2	2
**Setting**	**Urban/peri-urban**	6	6
	**rural**	9	9

Supplemental Table 2. Characteristics of health facilities by trial arm, India (N=30)

		Control (n=15)	Intervention (n=15)
**Facility level**	**Level I**	6	6
	**Level II**	8	8
	**Level III**	1	1
**Approximate births per year**	**≤300**	4	4
	**301-400**	6	6
	**401-1000**	4	4
	**>1000**	1	1

Supplemental Table 3. Participants by health facility, trial arm and country

Uganda (N=5102)				India (N=10,899)			
Control arm		Intervention arm		Control arm		Intervention arm	
Health centre	Participants enrolled	Health centre	Participants enrolled	Health centre	Participants enrolled	Health centre	Participants enrolled
1	525	2	437	2	307	1	455
3	306	4	438	4	223	3	775
5	130	6	219	5	214	7	458
7	100	8	157	6	495	9	401
9	123	11	324	12	138	10	345
10	249	12	99	16	145	11	220
16	167	13	123	19	541	13	377
19	89	14	145	20	120	14	262
20	125	15	140	22	378	15	288
21	85	17	107	23	381	17	399
22	80	18	49	24	276	18	379
25	102	23	96	26	570	21	199
27	100	24	188	27	223	35	428
28	129	26	111	29	659	28	513
30	99	29	60	31	509	30	221

Supplemental Table 4. Baseline characteristics of participants lost to follow-up

Characteristic	Uganda (N=5102) LTFU/missing N=1616 n (%)	Uganda (N=5102) Followed up N=3486 n (%)	p value	India (10,899) LTFU/missing N=1322 n (%)	India (10,899) Followed up N=9577 n (%)	p value
**Age (years)**			<0.001			0.77
**<20**	275 (17.0%)	416 (11.9%)		73 (5.5%)	511 (5.3%)	
**20-24**	724 (44.8%)	1,380 (39.6%)		488 (36.9%)	3,537 (36.9%)	
**25-29**	373 (23.1%)	979 (28.1%)		496 (37.5%)	3,594 (37.5%)	
**30-34**	155 (9.6%)	466 (13.4%)		201 (15.2%)	1,536 (16.0%)	
**35+**	89 (5.5%)	245 (7.0%)		64 (4.8%)	399 (4.2%)	
**Marital status**			<0.001			
**Married**	917 (56.7%)	1,566 (58.2%)		1,322 (100.0%)	9,577 (100.0%)	
**Cohabiting**	646 (40.0%)	1,041 (38.7%)				
**Not in a partnership**	53 (3.3%)	86 (3.2%)				
**Highest level of education (own)**			<0.001			0.074
**None or Primary School**	661 (40.9%)	978 (36.3%)		127 (9.6%)	746 (7.8%)	
**Secondary or High School**	863 (53.4%)	1,525 (56.6%)		847 (64.1%)	6,251 (65.3%)	
**Graduate or Postgraduate**	92 (5.7%)	190 (7.1%)		348 (26.3%)	2,580 (26.9%)	
**Highest level of education (partner)**			0.002			0.22
**None or Primary School**	246 (15.2%)	355 (13.2%)		266 (20.1%)	1,818 (19.0%)	
**Secondary or High School**	750 (46.4%)	1,244 (46.2%)		765 (57.9%)	5,405 (56.4%)	
**Graduate or Postgraduate**	160 (9.9%)	332 (12.3%)		182 (13.8%)	1,476 (15.4%)	
**Don't know**	407 (25.2%)	676 (25.1%)		109 (8.2%)	878 (9.2%)	
**N/a - no partner**	53 (3.3%)	86 (3.2%)				
**Perceived sufficiency of household income**			0.074			0.35
**Yes, it allows me/us to build savings**	84 (5.2%)	179 (5.1%)		76 (5.7%)	616 (6.4%)	
**Yes, it allows me/us to save a little**	350 (21.7%)	857 (24.6%)		81 (6.1%)	685 (7.2%)	
**Yes, it is just enough**	642 (39.7%)	1,403 (40.2%)		335 (25.3%)	2,258 (23.6%)	
**No, I/we must use savings**	325 (20.1%)	647 (18.6%)		597 (45.2%)	4,383 (45.8%)	
**No, I/we must borrow**	215 (13.3%)	400 (11.5%)		233 (17.6%)	1,635 (17.1%)	
**Occupation before pregnancy**			0.78			0.18

**Homemaker/Unemployed/Student**	949 (58.7%)	2,033 (58.3%)		1,046 (79.1%)	7,727 (80.7%)	
**In paid employment**	667 (41.3%)	1,453 (41.7%)		276 (20.9%)	1,850 (19.3%)	
**First antenatal care visit in months, mean (SD)**	3.57 (1.67)	3.55 (1.61)	0.62	2.37 (0.48)	2.44 (0.50)	<0.001
**Parity**			<0.001			0.005
**0**	630 (39.1%)	1,183 (33.9%)		898 (67.9%)	6,093 (63.6%)	
**1**	455 (28.2%)	964 (27.7%)		345 (26.1%)	2,688 (28.1%)	
**2**	261 (16.2%)	599 (17.2%)		75 (5.7%)	756 (7.9%)	
**3+**	266 (16.5%)	740 (21.2%)		4 (0.3%)	40 (0.4%)	

Supplemental Table 5. Additional pre-planned analyses for perinatal composite outcome

	Uganda						India					
	N	Prevalence control (95% CI)	Prevalence intervention (95% CI)	Prevalence difference (95% CI)	Prevalence ratio (95% CI)	ICC	N	Prevalence control (95% CI)	Prevalence intervention (95% CI)	Prevalence difference (95% CI)	Prevalence ratio (95% CI)	ICC
**Model 1**	3486	19.70 (17.86, 21.73)	20.04 (18.29, 21.95)	0.34 (-2.36, 3.03)	1.02 (0.88, 1.16)	0.000	9577	29.12 (27.42, 30.93)	30.19 (28.54, 31.94)	1.07 (-1.37, 3.51)	1.04 (0.95, 1.12)	0.003
**Model 2**	5102	19.73 17.97, 21.67)	19.83 (18.03, 21.82)	0.10 (-2.59, 2.80)	1.01 (0.87, 1.14)	0.000	10899	29.54 (27.91, 31.26)	30.57 (29.00, 32.23)	1.03 (-1.26, 3.33)	1.04 (0.96, 1.11)	0.002
**Model 3**	5102	19.65 (17.76, 21.74)	19.80 (17.94, 21.85)	0.15 (-2.69, 2.99)	1.04 (0.85, 1.22)	0.001	10899	29.43 (27.67, 31.31)	30.61 (28.91, 32.42)	1.18 (-1.32, 3.68)	1.04 (0.95, 1.13)	0.004
**Model 4**	5102	19.72 (17.99, 21.62)	19.83 (18.07, 21.76)	0.11 (-2.54, 2.75)	1.01 (0.87, 1.14)	0.000	10899	29.64 (28.00, 31.38)	30.48 (28.91, 32.13)	0.84 (-1.47, 3.15)	1.03 (0.95. 1.11)	0.002

Unless otherwise specified: all models intention-to-treat and adjusted for a priori cluster- and individual-level covariates
ICC: Intraclass correlation coefficient
Model 1: Complete case analysis
Model 2: Per protocol analysis
Model 3: Without adjustment for *a priori* individual level covariate
Model 4: With additional adjustment for unbalanced covariates at baseline[1]
[1]Uganda: history of non-live birth, family history of diabetes, previous hypertension, employment status, household income sufficiency, marital status, India: partner education status, timing of first ANC visit, history of non-live birth, previous macrosomia, previous hypertension, previous GDM

Supplemental Table 6. Cluster level summary analysis

	Uganda		India	
	N	β-coefficient (95% CI)	N	β-coefficient (95% CI)
**Perinatal composite outcome**	30	0.02 (-0.02, 0.06)	30	0.02 (-0.01, 0.05)

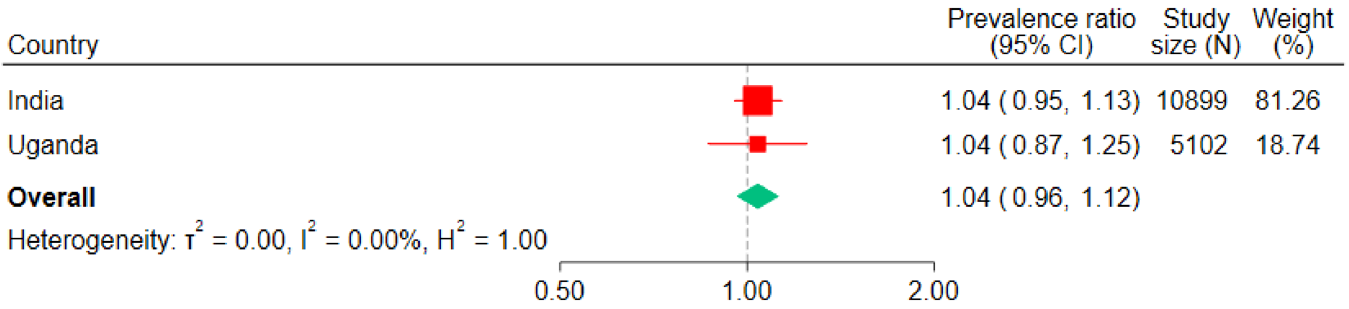

Supplemental Figure 1. Meta analysis without adjustment for *a priori* individual-level confounders (Model 3 in Supplemental Table 2)

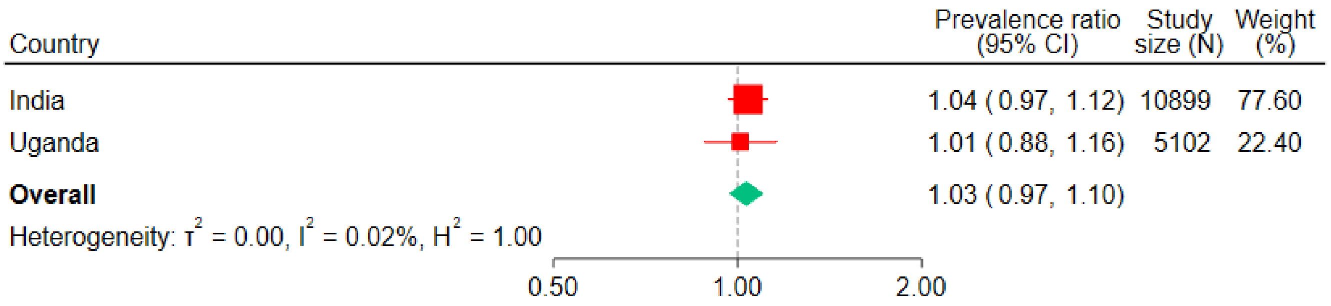

Supplemental Figure 2. Meta analysis for per protocol analysis (Model 2 in Supplemental Table 2)

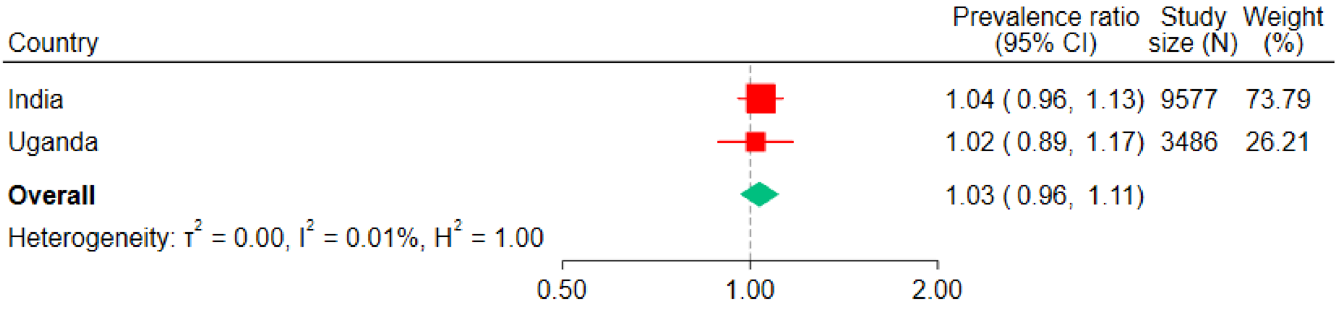

Supplemental Figure 3. Meta analysis for complete case analysis (Model 1 in Supplemental Table 2)

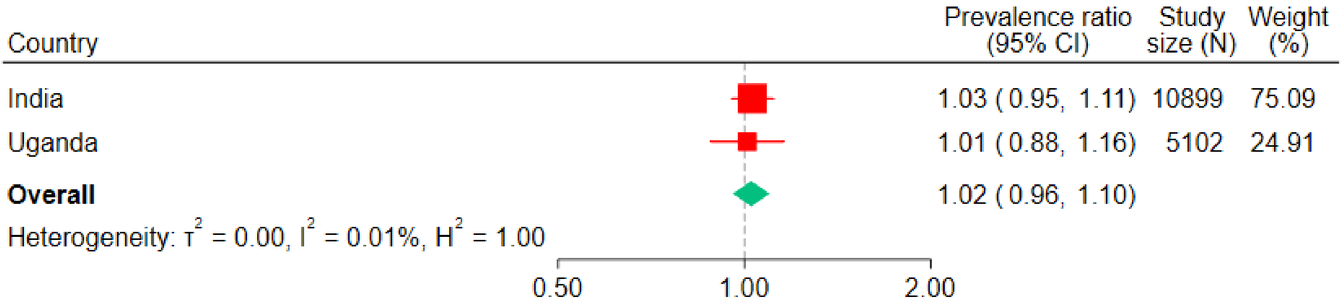

Supplemental Figure 4. Meta analysis with additional adjustment for individual-level covariates unbalanced at baseline (Model 4 in Supplemental Table 2)
