# Supplementary material for: Educational films for improving perinatal outcomes associated with gestational diabetes in Uganda and India: a cluster randomised trial
Source: BMJ Glob Health. 2026 Apr 27;11(4):e022676. doi: 10.1136/bmjgh-2025-022676 (PMC13141209; doi:10.1136/bmjgh-2025-022676)
Supplement: online supplemental file 2 [file bmjgh-11-4-s002.docx]

### BMJ Global Health Author Reflexivity Statement

Adapted from Morton, B., Vercueil, A., Masekela, R., Heinz, E., Reimer, L., Saleh, S., Kalinga, C., Seekles, M., Biccard, B., Chakaya, J., Abimbola, S., Obasi, A. and Oriyo, N. (2022), Consensus statement on measures to promote equitable authorship in the publication of research from international partnerships. Anaesthesia, 77: 264-276. <https://doi.org/10.1111/anae.15597>

| **Study conceptualisation** | |
| --- | --- |
| 1. How does this study address local research and policy priorities? | Gestational diabetes mellitus (GDM) is a priority issue in both India and Uganda. The Government of India has recognised GDM as an important cause of adverse pregnancy outcomes and published updated guidance on screening and management of GDM in 2018. In Uganda, it has been estimated that a high proportion of GDM cases are undiagnosed due to limited screening. Local research priorities have focused on improving screening and management, and the use of a film-based intervention potentially addresses the need for low-cost, inclusive and sustainable interventions. |
| 1. How were local researchers involved in study design? | This study was a partnership between research teams from India, Uganda and the UK. Researchers from each country were involved in seeking funding (SK, GB, MN, LO, JS, EON, and BS; 3 of 7 based in LMICs) and designing the study (LO, DR, AN, BS, HC, EON, JS, GB, MN, and SK; 5 of 10 based in LMICs) , and all subsequent study stages through to editing and approval the final manuscript. There is a senior academic acting as PI for each country (GB, MN, SK), and they are supported by a team of early-career and mid-career researchers from each country. Additionally, the UK PI’s (SK) research has been conducted predominantly in India and SE Asia, and he holds an adjunct position at the Public Health Foundation of India. Another senior co-author (JS) was based in Uganda for much of her research career. |
| **Research management** | |
| 1. How has funding been used to support the local research team(s)? | Project funding from the MRC/Newton Fund and DBT was used to cover salaries of the local research teams. |
| **Data acquisition and analysis** | |
| 1. How are research staff who conducted data collection acknowledged? | Research staff who coordinated and supervised data collection are included as co-authors. Additional fieldworkers and data collectors are acknowledged in the “Acknowledgements” section. |
| 1. How have members of the research partnership been provided with access to study data? | Country teams were responsible for supervising data collection and managing and cleaning study data. They had access to their own country-specific study data, with access to the data from the other country available on request. The UK analysts had access to both India and Uganda data. |
| 1. How were data used to develop analytical skills within the partnership? | Data analysis for the outcomes reported in this manuscript was conducted by UK researchers (LO, JL) with support from the wider team including a statistician (NB). Other team members in Uganda and India have led on analyses for other manuscripts using study data (e.g. qualitative analyses) and will have the opportunity to lead on further analyses. |
| **Data interpretation** | |
| 1. How have research partners collaborated in interpreting study data? | All research partners were involved as co-authors, and all participated in regular monthly team meetings during which findings were shared and interpreted. |
| **Drafting and revising for intellectual content** | |
| 1. How were research partners supported to develop writing skills? | Dara analysis and interpretation were conducted by early- and mid-career researchers, with support from senior co-authors to develop writing skills. |
| 1. How will research products be shared to address local needs? | This manuscript will be published as open access. Study teams in India and Uganda will share study findings across local networks. The intervention study films will be made freely available via Medical Aid Films. |
| **Authorship** | |
| 1. How is the leadership, contribution and ownership of this work by LMIC researchers recognised within the authorship? | The study team is a partnership of researchers from low-income (Uganda), middle-income (India) and high-income (UK) countries. The PIs from each of the three countries are joint last authors, and the joint first authors are from the middle- and high-income research teams. Of the 19 co-authors, 9 are from LMICs (DR, AN, YA, BS, MK, EL, DM, MN, GRB). |
| 1. How have early career researchers across the partnership been included within the authorship team? | 9 of the 19 co-authors are early career researchers (pre-PhD or early postdoctoral at the time the study was started) (DR, AN, YA, PM, MK, EL, NB, LH and JL). Five of these 9 are from LMICs (DR, AN, YA, MK, EL). |
| 1. How has gender balance been addressed within the authorship? | 11 of 19 co-authors are female, including the three joint first authors. |
| **Training** | |
| 1. How has the project contributed to training of LMIC researchers? | The study has provided training for early career researchers across all three research teams (Uganda, India, UK), with a focus on capacity building. The India and Uganda teams have led manuscripts on qualitative findings from the study (and are likely to lead future manuscripts), submitted abstracts for academic meetings and conferences, and presented details of the study methodology and findings at within-country events and meetings. |
| **Infrastructure** | |
| 1. How has the project contributed to improvements in local infrastructure? | The study has contributed to local infrastructure through the use of local film crews for producing the intervention films (these were all shot on location in India and Uganda) and the use of a local company to develop the data collection app in India.  Research partnerships have been forged and strengthened and have continued beyond the study period. |
| **Governance** | |
| 1. What safeguarding procedures were used to protect local study participants and researchers? | Ethical approval was obtained from relevant ethical committees in the UK and the two research settings (India and Uganda). All study procedures followed good clinical practice and LSHTM standard operating procedures. Data were anonymized before being shared with the UK team who conducted data analysis. During the Covid pandemic, the trial was adapted to protect participants and research staff from unnecessary exposure to Covid-19. |
